# Ethanol-Induced Neurodegeneration and Glial Activation in the Developing Brain

**DOI:** 10.3390/brainsci6030031

**Published:** 2016-08-16

**Authors:** Mariko Saito, Goutam Chakraborty, Maria Hui, Kurt Masiello, Mitsuo Saito

**Affiliations:** 1 Division of Neurochemisty, Nathan S. Kline Institute for Psychiatric Research, 140 Old Orangeburg Rd., Orangeburg, NY 10962, USA; chakrabortyg@aol.com (G.C.); mhui@nki.rfmh.org (M.H.); kmasiello@nki.rfmh.org (K.M.); 2 Department of Psychiatry, New York University Langone Medical Center, 550 First Avenue, New York, NY 10016, USA; mitsaito@nki.rfmh.org; 3 Division of Analytical Psychopharmacology, Nathan S. Kline Institute for Psychiatric Research, 140 Old Orangeburg Rd., Orangeburg, NY 10962, USA

**Keywords:** ethanol, fetal alcohol spectrum disorders, neurodegeneration, glial activation, neuroinflammation, microglia, astrocytes, lipopolysaccharides, developing brain, phagocytosis

## Abstract

Ethanol induces neurodegeneration in the developing brain, which may partially explain the long-lasting adverse effects of prenatal ethanol exposure in fetal alcohol spectrum disorders (FASD). While animal models of FASD show that ethanol-induced neurodegeneration is associated with glial activation, the relationship between glial activation and neurodegeneration has not been clarified. This review focuses on the roles of activated microglia and astrocytes in neurodegeneration triggered by ethanol in rodents during the early postnatal period (equivalent to the third trimester of human pregnancy). Previous literature indicates that acute binge-like ethanol exposure in postnatal day 7 (P7) mice induces apoptotic neurodegeneration, transient activation of microglia resulting in phagocytosis of degenerating neurons, and a prolonged increase in glial fibrillary acidic protein-positive astrocytes. In our present study, systemic administration of a moderate dose of lipopolysaccharides, which causes glial activation, attenuates ethanol-induced neurodegeneration. These studies suggest that activation of microglia and astrocytes by acute ethanol in the neonatal brain may provide neuroprotection. However, repeated or chronic ethanol can induce significant proinflammatory glial reaction and neurotoxicity. Further studies are necessary to elucidate whether acute or sustained glial activation caused by ethanol exposure in the developing brain can affect long-lasting cellular and behavioral abnormalities observed in the adult brain.

## 1. Introduction

**Figure 1:**
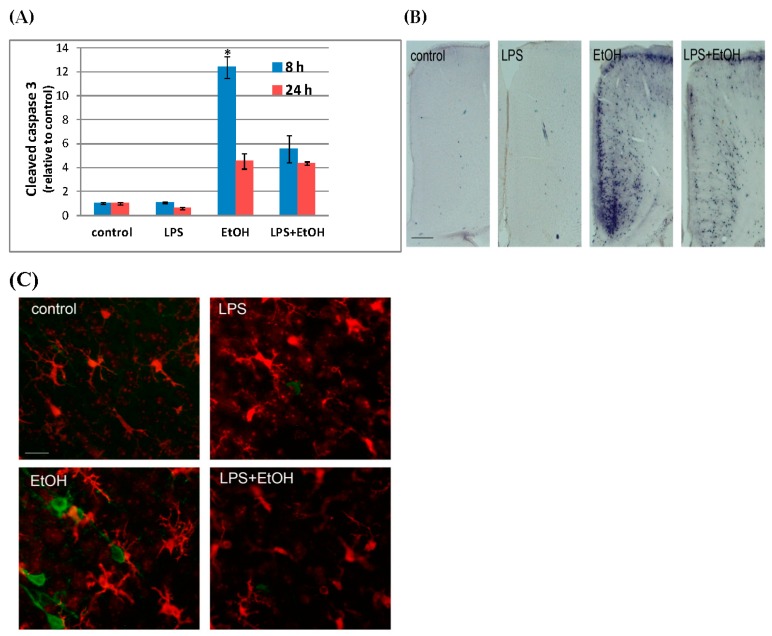
LPS attenuates P7 ethanol-induced caspase-3 activation and morphological changes in microglia. (**A**) LPS (0.5g/kg)/saline was injected (i.p.) into P7 mice 2 h before ethanol (EtOH) (2.5g/kg, twice, 2 h apart)/saline injection, and 8 h and 24 h after the first ethanol injection, forebrains were taken and homogenates were analyzed by Western blots. The content of CC3 was normalized by actin and expressed as the ratio to the control. * Significantly different from all other groups by the Bonferroni post-hoc test after one-way ANOVA for 8 h samples; (**B**) P7 mice were treated as described in A, and 8 h after the first ethanol injection, mice were perfusion-fixed and brain sections were stained using anti-CC3 antibody. The representative images show the cingulate cortex region, and the bar indicates 200 µm; (**C**) Brain sections prepared as described in B were dual-labeled with anti-Iba1 (red) and anti-cleaved tau (green) antibodies. The bar indicates 20 µm.

**Figure 2:**
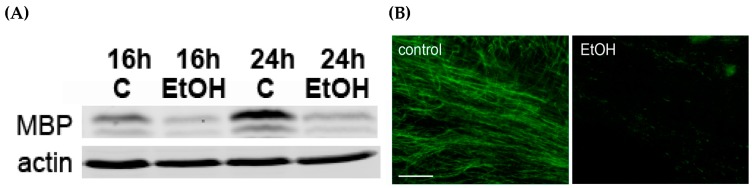
P7 ethanol reduces MBP content. (**A**) 16 and 24 h after P7 saline/ethanol (EtOH) injection, MBP in the forebrain homogenates was analyzed using anti-MBP antibody (Santa Cruz) by Western blot; (**B**) 48 h after saline/ethanol injection at P7, mice were perfusion-fixed, and the brain sections were labeled using anti-MBP antibody. The representative images show the corpus callosum/cingulum regions. The bar indicates 50 µm.

Prenatal ethanol exposure affects fetal development especially in the brain, leading to fetal alcohol spectrum disorders (FASD) with a range of mild to severe symptoms [1]. The incidence of FASD is approximately 2–5 in 100 births, and FASD are the leading cause of mental retardation in the United States [2,3]. FASD neuropathology includes volume reduction in the corpus callosum, cerebral cortex, cerebellum, and subcortical structures such as the hippocampus, basal ganglia, amygdala, and thalamus; the cognitive consequences of FASD include deficits in executive function, learning and memory, and information processing [1,4,5,6]. Mechanisms of FASD have been studied using various animals prenatally or neonatally exposed to ethanol, because these animal models display some of the structural and behavioral abnormalities observed in human FASD. It has been predicted that ethanol affects every step of brain development including proliferation, differentiation, and migration of neural cells. For instance, binge-like ethanol exposure in early postnatal rodents during the period equivalent to the third trimester of human pregnancy induces robust neurodegeneration [7,8,9,10,11], because neurons are particularly vulnerable to environmental toxins during this period of heightened synaptogenesis [12]. This ethanol-induced neurodegeneration, which is associated with glial activation [13,14,15,16,17,18,19] and elevation of pro- and anti-inflammatory cytokines [13,20,21,22,23], may result in long-lasting cellular, physiological, and neurobehavioral deficits [24,25,26,27,28,29,30,31,32]. It is conceivable that ethanol-induced glial activation/neuroinflammation in animal models of FASD is involved in the long-lasting brain damage, as reported in various neurodegenerative diseases [33] and brain injuries [34]. Ethanol is known to affect immune activity in the brain throughout the life span [35]. In adult animals, ethanol increases the expression of proinflammatory cytokines and chemokines as well as molecules such as nitric oxide and cyclooxygenase-2, which may lead to neurodegeneration [36,37,38]. These inflammatory mediators are produced by activated microglia and astrocytes [36,37,39], although some may be derived from the blood [40], because ethanol administration induces systemic inflammation through multi-organ interactions [41]. In particular, microglia can be activated through their receptors (such as Toll-like receptor 4 (TLR4)) directly by ethanol or indirectly by secondary stimuli [36,42]. Then, the activated microglia present either proinflammatory M1-like phenotype (producing proinflammatory and cytotoxic mediators), anti-inflammatory M2-like phenotype (enhancing clearance of debris and producing anti-inflammatory mediators), or intermediate phenotypes [34,43,44,45], and the imbalance between these phenotypes can lead to neuroinflammation and neurodegeneration. Ethanol-induced neurodegeneration, glial activation, and the production of inflammatory mediators have also been shown in the developing brain. However, the relationship between neurodegeneration and glial activation is likely to be different from that of the adult brain. This review focuses on how microglia and astrocytes are involved in neurodegeneration in third-trimester binge-drinking rodent models of FASD. In Section 2, the general relationship between neurodegeneration and glial activation/neuroinflammation is summarized, including inflammation induced by lipopolysaccharide (LPS), an agonist of TLR4. Then, the effects of ethanol on neurodegeneration/neuroinflammation in vitro and in the adult brain are discussed in Section 3. In Section 4, ethanol-induced neurodegeneration/neuroinflammation in the developing brain is described, and in Section 5, studies (including our new experimental results (Figure 1 and Figure 2)) about the comparison between the effects of LPS and ethanol in the neonatal brain are summarized.

## 2. Neurodegeneration Is Tightly Associated with Glial Activation

Neurodegeneration seems to be always associated with glial activation. While glial activation can be an important step to protect the brain from harmful endogenous and exogenous stimuli including infectious organisms and degenerating neurons [34], sustained and/or uncontrolled neuroinflammatory responses involving microglia and astrocytes contribute to disease progression including neurodegeneration in animal models of neurodegenerative diseases [33] and brain injuries [34]. Specifically, microglia, the resident macrophages, are considered the primary cell type of such innate immune systems in the brain [46]. Depending on environmental signals, microglia acquire different phenotypes that are often classified using nomenclatures of macrophages: classical proinflammatory (M1), alternate anti-inflammatory (M2), and intermediate phenotypes [34,43,44,45], although this classification is only one of the designation systems for characterizing functions of activated microglia [34] and may not accurately represent complex microglial polarization, which is different from that of macrophages [47]. It is thought that acute brain injury such as traumatic brain injury and ischemic reperfusion injury triggers proinflammatory microglial activation, which can lead to secondary brain damage including neurodegeneration [48]. Initially, acute brain injury releases danger signals called damage-associated molecular patterns (DAMPs), which include chaperone proteins, high-mobility group protein B1 (HMGB1), nucleic acids, and nucleotide derivatives. DAMPs, which also include pathogen-associated molecular patterns (PAMPS) derived from endogenous and exogenous pathogens, activate pattern recognition receptors such as TLRs on microglia [34,48,49]. TLRs regulate signal transcription factors (e.g., nuclear factor kappa b (NF-κB), activator protein-1 (AP-1), and the interferon-regulatory factor (IRF) family) in response to DAMPs and enhance the expression of proinflammatory and cytotoxic factors (e.g., tumor necrosis factor alpha (TNFα), interleukin-1 beta (IL-1β), TLRs, cytokine receptors, and reactive oxygen species (ROS)), which may lead to secondary brain damage [48,50,51]. Activation of TLR4 on microglia by an agonist LPS, a PAMP derived from Gram-negative bacteria, has been widely used to examine the proinflammatory pathway in M1-like microglia [52,53]. Microglia are the major TLR4 expressing cells in the CNS, although astrocytes and oligodendrocytes also express TLR4 to some extent [52], and astrocytes activated by LPS can produce increased levels of proinflammatory cytokines [44,45]. LPS-activated TLR4 on microglia triggers the myeloid differentiation factor 88 (MyD88)-dependent pathway and TIR-domain-containing adapter-inducing interferon-β (TRIF)-dependent pathway. MyD88-dependent pathway induces mitogen-activated protein kinase (MAPK) and NF-κB, and the TRIF-dependent pathway generates interferon (IFN)-β and IFN-inducible genes; both signaling pathways lead to the elevation of various immune and inflammatory genes [54]. In vitro, LPS exposure alone or in combination with IFN-γ triggers microglial activation, leading to massive production of proinflammatory and cytotoxic factors such as TNFα, IL-6, and nitric oxide, which can induce neuronal death [51,53,55]. However, neurons are resistant in the absence of functional TLR4, indicating a relationship between innate immunity and neurodegeneration [53]. While LPS entrance into the adult brain is low [56], acute systemic LPS injection into rodents also induces microglial activation including production of TNFα, although neuronal death occurs only in certain neuronal cell types [46,57,58]. TNFα increased by LPS may remain elevated in the brain for months and may exert prolonged detrimental effects on the brain [58]. 

Thus, while M1-like microglia is proinflammatory and can be neurotoxic, M2-like microglia may enhance debris clearance and suppress neuroinflammation by producing anti-inflammatory mediators including IL-10, IL-4, and transforming growth factor (TGF-β) [34,59]. Activated microglia phagocytose dead neurons, apoptotic bodies [60,61,62], and synapses [59]. Apoptotic cells can be efficiently engulfed by microglia through ‘find-me’ signals such as lysophosphatidylcholine, sphingosine 1-phosphate, and fractakine (CX3CL1), and ‘eat-me’ signals such as phosphatidylserine (PS) in the outer plasma membrane of apoptotic cells [63], and microglia have PS receptors and bridge proteins necessary for phagocytosing apoptotic neurons [64]. Once engulfed by microglia, the efficient digestion of apoptotic cells prevents further neurodegeneration. While phagocytosis of pathogens requires initiation of an inflammatory response, removal of apoptotic cells is considered a process that prevents excessive inflammation [65], and this type of phagocytosis of apoptotic cells, which occurs in an ‘immunologically silent’ manner, is classified as efferocytosis [66]. Thus, the balance between M1 and M2 microglia, which is regulated by signals from endogenous or exogenous origins, would determine whether glial activation leads to secondary neurodegeneration/chronic neuroinflammation or neuroprotection/resolution of neuroinflammation [43,45,48]. 

In addition to microglia, astrocytes are also involved in neurodegeneration/inflammation. Many kinds of brain insults trigger astrocyte activation (astrogliosis) [44], which is associated with hypertrophy, upregulation of intermediate filaments (nestin, vimentin, glial fibrillary acidic protein (GFAP)), and cell proliferation [49,67]. The degree of astrogliosis varies from mild/moderate astrogliosis, recognized by upregulation of GFAP and hypertrophy, to severe astrogliosis, which leads to astrocyte proliferation and glial scar formation [68]. Molecules that induce initial astrogliosis include TNFα and IL-1β released from activated microglia and nucleosides and HMGB1 generated in the injury sites [48,67]. Once activated, astrocytes exert both neuroprotective and neurotoxic effects, as observed in activated microglia. While microglia are thought to be the major innate immune cells and the primary source for cytokine production [46], activated astrocytes also enhance the expression of proinflammatory and neurotoxic mediators such as IL-1β, TNFα, and IL6 [67], chemokines, neurotoxic levels of ROS, and excitotoxic glutamate, and compromise blood–brain barrier functions [49,68]. On the other hand, reactive astrocytes can produce factors to support repair and regeneration after brain damage [49,68] and also engulf whole dead cells in brain injury [69]. Glial scar formation is believed to prevent the progression of tissue damage, but inhibits axonal regeneration [70], although a recent report indicates that scar formation may aid CNS axon regeneration [71]. 

In contrast to acute neuroinflammation, chronic neuroinflammation shows long-lived and persistent inflammatory responses, which may be caused by uncontrolled dynamics between pro- and anti-inflammatory microglia and astrocytes [34]. Proinflammatory factors such as TNFα and IL-1β produced by microglia may act directly on neurons to induce apoptosis [72,73], but also activate astrocytes that release toxic mediators including nitric oxide and ROS, and can damage the surrounding tissue [74]. Then the released DAMPs from the damaged tissue can further increase inflammation and glial activation, leading to a vicious inflammatory cycle [34,49]. Also, the synthesis and release of cytokines and chemokines lead to persistent migration of monocytes and neutrophils across the blood–brain barrier in some cases [75]. Those myeloid cells may be pro- or anti-inflammatory depending on the type of injury [76].

In summary, these studies indicate that degenerating neurons cause glial activation through the release of DAMPs and/or expression of “find-me” and “eat-me” signals, resulting in neuroinflammation and/or neuroprotection. Escalated or prolonged neuroinflammation may cause neurodegeneration by pro-inflammatory and neurotoxic factors released by activated glia and migrated myeloid cells. 

## 3. Excessive Ethanol Intake May Induce Neurodegeneration and Glial Activation/Neuroinflammation 

Alcoholism is known to cause neurodegeneration/neuroinflammation, which has been proposed as one of the alcoholism-induced neuropathological mechanisms [39,40,77]. Human and animal studies indicate that excessive ethanol intake induces damage in the brain including neurodegeneration during various developmental stages [38,78,79,80], and such ethanol-induced neurodegeneration appears to be associated with glial activation and neuroinflammation [36,80]. Studies using MRI have shown that brain structural changes associated with alcoholism are enlargement of the ventricles and shrinkage of the frontal cortex, the underlying white matter, and the cerebellum [38,81,82]. These MRI studies agree with postmortem studies showing white matter changes as well as atrophy of neurons in brain regions such as the cortex and the cerebellum [38,79,81]. In parallel with brain atrophy, alcoholic brains show signature of glial activation/neuroinflammation. Human alcoholics have increased levels and/or expression of immune genes such as TLR2, TLR3, TLR4, and HMGB1 [42], HMGB1 receptor RAGE (receptor for advanced glycation end-products) [83], NF-κB [84], IL-1β and inflammasome proteins [85], monocyte chemoattractant protein-1 (MCP-1), and microglial markers [86] in addition to histochemical markers of neuronal cell death [39]. Adult rodents administered with binge levels of ethanol for four days (the Majchrowicz model [87]) display neurodegeneration with necrotic appearances in the several cortex regions and hippocampal dentate gyrus [38,77,81,88,89,90], and show elevation of oxidative stress, increased NF-κB, decreased cAMP response element binding protein (CREB) expression [38], increased neuroinflammation-linked enzymes, such as phospholipase A2 [90], and astrocyte activation [77]. However, the treatment induces only a low level of M1-like microglia or induces M2-like microglia judged by cytokine profiles [91,92]. If such four-day binge drinking is repeated after a seven-day recovery period, an increased level of TNF-α and greater expression of Iba-1 and OX42 immunoreactivity are observed, indicating the presence of M1-like microglia [93]. Similarly, 10 days of binge ethanol administration inducing neurodegeneration [39] increases prolonged expression of a variety of neuroimmune genes, including TNFα, IL-1β, and MCP-1 in the brain [94], although the immune response seems to be region-specific [35]. While these brain cytokines and chemokines may be partially derived from the blood [40], because ethanol administration induces systemic inflammation through multi-organ interactions [41], the elevated cytokines in the brain seem to remain longer compared to those in the plasma and the liver [94]. Also, chronic ethanol treatment (for five months) of adult rats induces the release of pro-inflammatory mediators, astrocytic and microglial activation, and caspase-3 activation in the cerebral cortex [95]. In contrast, acute (one day) ethanol treatment induces no or very low levels of neurodegeneration only in the specific brain regions [89,96] and no significant changes in the protein levels of TNFα and MCP-1 [94], although it induces reactive gliosis detected by increases in vimentin [96]. Also, acute ethanol treatment in slice culture barely increases TNFα and IL-1β [97], and even suppresses TNFα, IL-1β, and IL-6 immune responses to LPS [98] in both in vivo and in vitro models, probably by attenuating TLR4 signaling [80]. These studies suggest that acute or short-term ethanol exposure barely induces M1-like microglia activation, while longer or repeated ethanol exposure induces M1-like microglia activation. Regarding the mechanisms of neuroinflammation, the 10 daily doses of ethanol also increase TLR2, TLR3, TLR4, and HMGB1 expression [42] as seen in human alcoholics, and the brain slice culture experiments indicate that neutralizing antibodies to HMGB1 or siRNAs against HMGB1 or TLR4 attenuates the induction of IL-1β by ethanol [42]. These results suggest that ethanol induces neuroimmune activation and the production of cytokines through the HMGB1/TLR signaling in the brain [42] in addition to the possible elevation of cytokines derived from the blood [40]. It is indicated that decreased HDAC activity by ethanol releases neuronal HMGB1, which activates TLR4 and leads to inflammatory cascades [99]. Also, chronic (five months) ethanol treatment-induced neurodegeneration and proinflammatory microglial activation [95] are inhibited by TLR4 deficiency, implying that TLR4 is involved in both ethanol-induced glial activation and neurodegeneration [36] and raising the possibility that neurodegeneration is a consequence of glial activation. However, in the four-day binge drinking model, the time course of expression of microglial markers and neuronal death supports the notion that ethanol-induced microglial activation is a consequence of neurodegeneration [93,100]. 

Cultured cells have also been used to examine mechanisms of ethanol-induced neuroinflammation and neurodegeneration. In general, the effects of ethanol on neuroimmune gene expression are similar to the effects of LPS or IL-1β, although ethanol induces a much smaller response [101]. The addition of ethanol in the microglial culture changes their shapes and induces pro- and anti-inflammatory mediators, indicating that ethanol directly activates microglia, and such an ethanol-treated microglial culture medium enhances ethanol-induced oxidative stress and apoptosis in cultured hypothalamic neurons [102], suggesting that neuroinflammation at least partially contributes to ethanol-induced neuronal death. The effects of ethanol on the activation of the MAPK and NF-κB pathways and the production of inflammatory mediators are not observed in cultured microglia or astrocytes prepared from TLR4 knockdown mice [36,103], underscoring the importance of TLR4 in ethanol-induced glial activation. 

In summary, studies in adult rodents indicate that under chronic or semi-chronic ethanol treatment conditions, inflammatory mediators elevate in the brain. Some of these mediators may be transferred from the blood into the brain. However, ethanol seems to activate TLR4 on microglia (and perhaps astrocytes) directly or indirectly through secondary stimuli such as HMGB1 released from neurons. Activation of the TLR4/MyD88-dependent and/or independent pathways on microglia (astrocytes) stimulates NF-κB and AP-1, which promote the expression of innate immune cytokines as well as of TLRs and cytokine receptors. Ethanol-released HMGB1 may also bind to RAGE (one of the HMGB1 receptors) on microglia and stimulate the NF-κB pathway. The resultant elevation of proinflammatory and neurotoxic mediators, if excessive or prolonged, seems to induce neurodegeneration. However, in the adult brain treated with acute ethanol, M1-like glial activation seems to be limited by M2-like glial formation, leading to resolution of neuroinflammation, and also the levels of neurodegneration is very low. On the other hand, acute ethanol exposure in the neonatal rodent brain causes profound neurodegeneration as described in Section 4 and Section 5, suggesting age-dependent differences in neuronal and glial responses to ethanol.

## 4. Glial Activation Is Associated with Neurodegeneration in Animal Models of FASD

The developing brain is especially vulnerable to ethanol toxicity, and prenatal alcohol exposure causes FASD, which manifest long-lasting structural and functional brain abnormalities [1]. MRI studies indicate that FASD patients show a reduction in the cranial vault and the corresponding reduction in the overall size of the brain and the cerebellum [104]. Also, reduced gyrification of the cortex [105] and a reduction in the surface area of the anterior cingulate cortex are observed among adolescents with heavy prenatal alcohol exposure [106]. The post mortem pathology of FASD includes microcephaly and volume reduction in the corpus callosum, cerebral cortex, cerebellum, and subcortical structures including the hippocampus, basal ganglia, amygdala, and thalamus [1,4,5,6]. The cognitive consequences of FASD include deficits in executive function, learning and memory, information processing, vigilance, mathematical ability, speech and language skills, and visual/spatial ability [1,6]. Considering such long-lasting damage to the developing brain triggered by prenatal ethanol and the tight relationship between ethanol and glial activation/neuroinflammation, one can imagine that glial activation may be linked to neurodegeneration and play an important role in the development of FASD [16,107,108]. Although data are still scant, some studies aim to address roles of glial activation/neuroinflammation in ethanol-induced damage in the developing brain using animal models of FASD. It has been shown that cortical volume, thickness, and surface area are reduced by ethanol exposure throughout gestation in rodents [1,109]. It is known that ethanol affects every step of brain development, including the proliferation, differentiation, and migration of neural cells. For example, ethanol exposure during gestation day 11–21 rats causes decreased neurogenesis, disrupted radial glia, and reduced migration and survival of neurons in the neocortex, hippocampus, and principal sensory nucleus of the trigeminal nerve, resulting in a permanent abnormal organization of the cerebral cortex [110]. Exposure to ethanol during the early postnatal period in rodents (equivalent to the third trimester of gestation in humans) induces apoptotic cell death in many brain regions, microcephaly, and cerebellar abnormalities [10,11,111,112,113,114]. Such ethanol-induced brain damage in neonates results in long-lasting brain structural [24,25,27,29] and behavioral abnormalities [9,31,32]. Even one day of acute ethanol exposure, which induces robust apoptosis [10,11], results in abnormalities in cellular, structural, physiological, and behavioral levels in the adult brain [25,26,27,29,30,31,32].

There are several reports indicating that such ethanol-induced acute and/or long-lasting neural damage in the third-trimester models of FASD is associated with glial activation and neuroinflammation. It has been reported that ethanol causes death of Purkinje neurons and microglia in the cerebellum in mice treated from P3 to P5 (by gavage at 3.5 g/kg body weight), while microglia that survive the toxic effects of ethanol show a proinflammatory phenotype [16]. Also, ethanol treatment of mice (P4-9, by gavage at 4 g/kg) induces morphological activation of microglia at P10 and increases expression of IL-1β and TNFα mRNA in the hippocampus, cerebellum, and cerebral cortex, and chemokine (C-C motif) ligand 2 (CCL2) mRNA in the hippocampus and cerebellum [20]. Furthermore, these effects are blocked by an anti-inflammatory peroxisome proliferator-activated receptor (PPAR)-γ agonist [20]. Ethanol injection into neonatal mice (P4-9, by intraperitoneal (i.p.) injection. 2.2 or 4.4 g/kg) or one-time injection (i.p. 4.4 g/kg) at P4 induces immediate death of granule and Purkinje neurons in the cerebellum, which is associated with activation of dsRNA-activated protein kinase (PKR) and production of IL-1β, a down-stream target of PKR [21]. Furthermore, deletion of PKR inhibits cell death as well as IL-1β production, suggesting that neuroinflammation may be related to ethanol-induced neurodegeneration [21]. A rat study shows that ethanol treatment (P4-9) induces microglial morphological activation and elevation in expression of CCL4 and TGF-β in the hippocampus at P10, although TNFα and IL-1β increase in both ethanol-treated and sham-intubated animals [115] in this study. While a low concentration of ethanol vapor exposure in rats from P2 to P16 does not induce elevation of proinflammatory factors (IL-1β, MCP-1) [116], a higher concentration of ethanol vapor inhalation from P3 to P5 increases levels of IL-1β, TNFα, and TGF-β, and the number of GFAP-positive (GFAP^+^) cells in both the cerebellum and the hippocampus at P6, although neurodegeneration and morphological activation of microglia are only seen in the cerebellum [23]. In these experiments, while Purkinje cell loss is observed even at P45, cytokine and chemokine elevation occurs only transiently during the neonatal period [23], although another study shows that rats treated with ethanol (5 g/kg, by intragastric intubation, P7-9) are associated with increased cytokine (TNFα, IL-1β, and TGF-β ) levels in the cerebral cortex and hippocampus at P28 [22]. Similar to the effects of the repeated ethanol administration, a one-day acute binge on ethanol at P7 induces robust neurodegeneration of various types of neurons (such as pyramidal cells) in many brain regions, including specific layers of cortices, many nuclei of the thalamus, the caudate nucleus, and the hippocampus [11]. The acute neuronal death induced by P7 ethanol is mitochondria-mediated apoptosis, involving Bax activation, cytochrome c release, and caspase-3 activation, which occurs 6–8 h after ethanol exposure; this is followed by neurodegeneration detected by amino cupric silver or Fluoro-Jade stain around 16–24 h [11,31,117]. Our immunohistochemical studies indicate that subcutaneous injection of ethanol in P7 mice (2.5 g/kg, 2 h apart, twice) induces not only neurodegeneration in various brain regions as described above but also activation of microglia judged by their morphology [17,18,19]. Microglia change their shapes from the resting (a small cell body and long branches) to partially activated state (bigger cell body and thicker branches), detected by Iba1 (ionized calcium-binding adapter molecule 1, specific to microglia and macrophages) immunostaining within 4 h after the first ethanol injection, and then change to rod- or amoeba-like shapes within 24 h [17,19]. These activated microglia are localized in the same area where apoptotic neurodegeneration is detected by Fluoro-Jade stain, such as in layers IV/V of the cortex, and Fluoro-Jade stain is frequently observed in Iba1-positive microglia [19]. In addition, the rod- or amoeba-shaped microglia are strongly labeled with antibody against CD68, a marker for phagocytes [19], and these microglia are labeled with antibody against cleaved tau (tau cleaved by activated caspase-3) present in degenerating neurons [17], suggesting that these microglia are phagocytes engulfing degenerating neurons. The activated microglia specifically express GM2 ganglioside, which is barely seen in the control brain [18,19]. GM2 may be derived from degenerating neurons because neurons are enriched in highly glycosylated gangliosides, which can be degraded to GM2 in lysosomes. However, the presence of phagocytes engulfing degenerating neurons is transient, and microglia are back to the original resting shape within 48 h [18,19]. Ahlers and his colleagues [13] also show that P7 ethanol activates GFP-labeled microglia in CX_3_CR1^gfp^ mice, and microglial activation is detected in the neocortex where neurodegeneration occurs. These activated microglia contain markers of late-stage apoptotic neurons and apoptotic bodies (PS binding dye) and are deactivated within 1–2 days. The authors show further that ethanol-induced microglial activation and transient elevation of TNFα and IL-1β are largely abolished in BAX null mice lacking apoptotic neurodegeneration. These studies indicate that the major initial responses of microglia are toward apoptotic neurons, which express “find-me” signals (such as nucleotides) and “eat-me” signals (such as PS) in the outer plasma membrane [63]. Microglia recognize the signals through molecules such as PS receptors [64], and engulf and digest apoptotic neurons by efferocytosis to prevent further neurodegeneration [65,66,118]. However, acute ethanol-induced transient elevation of TNFα and IL-1β [13] indicates that microglia show not only M2 phenotypes but also M1 phenotypes. It may be because some “find-me” signals increase both pro- and anti-inflammatory factors [63]. Also, activation of PKR shown in P4 mice treated with ethanol [21] may indicate that TLRs on microglia are also activated by ethanol, and the resultant elevation of proinflammatory cytokines may be involved in the process of neurodegeneration. 

In addition to microglia, astrocytes are also affected by ethanol in the developing brain. Prenatal ethanol exposure decreases GFAP expression in early postnatal rats [119], while neonatal ethanol exposure leads to an increase in GFAP expression [14,120]. In the neonatal brain, the increase in GFAP expression is rather confined to specific areas, suggesting that this is caused by increased neuronal damage or the release of proinflammatory cytokines by microglia [14]. Our experiments show that acute P7 ethanol induces not only microglial activation, but also astrocyte activation detected by elevation of GFAP-positive (GFAP^+^) astrocyte densities [19]. The increased GFAP^+^ astrocytes are present in the area where neurodegeneration occurs, such as in layers IV/V of the cortex, as well as in the deep cortical layer/white matter near the cingulum and external capsule, and these astrocytes show slight hypertrophy. The densities of GFAP^+^ astrocytes increase 12–24 h after the appearance of phagocytic microglia [19], and the elevation is still significant at P14 (manuscript in preparation). These increased GFAP^+^ astrocytes express transient GM2 ganglioside expression as observed in activated microglia. It is still unknown whether such astroglial activation by P7 ethanol is neuroprotective or neurotoxic, or proinflammatory or anti-inflammatory. 

## 5. Comparison between the Effects of LPS and Ethanol on Neurodegeneration and Glial Activation in the Neonatal Brain

To further explore characteristics of neurodegeneration and glial activation caused by acute ethanol treatment in the neonatal brain, the comparison of the effects of LPS and ethanol can be useful, because both LPS and ethanol activate glia through TLR4, as described in the previous sections. It has been shown that the systemic LPS injection (2 mg/kg i.p.) into P5 rats induces apoptosis of oligodendrocytes, activation of microglia and astrocytes, elevation of TNFα and IL-1β, and injury to dopaminergic neurons [121]. Although LPS entrance into the brain may be low, systemic LPS appears to elicit TLR4 signaling in the brain independent of peripheral cytokine responses [122]. Acute intracerebral injection of LPS (1 mg/kg) into P5 rats similarly induces loss of oligodendroglial progenitor cells and apoptosis of oligodendrocytes in the cingulum area, hypomyelination, activation of microglia and astrocytes, and elevation of IL-1β and TNFα [123,124], and the inhibition of microglial activation can protect periventricular developing oligodendrocytes from LPS injury [125]. Some of the changes induced by intracerebral LPS injection into P5 rats, such as increased number of activated microglia, elevation of IL-1β, and the damage to dopaminergic neurons, are still observed in the P70 brain [124,126]. Also, LPS administration (1 mg/kg, i.p.) at P9 specifically affects the survival of dividing neurons and astrocytes in the dorsal hippocampus [127]. Compared to a single LPS application, repeated systemic challenge with LPS may sustain the M1 microglial state and trigger the loss of neurons. LPS administration (i.p., 6 mg/kg) from P4 to P6 induces acute astrogliosis, microgliosis, and neuronal loss in the pons, cerebellum, and hippocampus, and increases HMGB1, TLR4, and matrix metallopeptidase 9 expression levels [128]. However, depending on the dose of LPS, repeated LPS administration often causes LPS hyporesponsiveness/endotoxin tolerance [129]. Related to this, LPS shows neuroprotection against the subsequent neuronal insult (LPS preconditioning). For instance, a peripheral injection of 0.05–1.0 mg/kg LPS into adult rodents can induce transient neuroprotection against ischemia, stroke, and more destructive LPS treatments [130,131,132]. The rapid tolerance may be achieved by direct interference with the TLR/cytokine signaling pathways, and the delayed tolerance may include upregulation of feedback inhibitors of inflammation such as anti-inflammatory cytokines [130,133]. It has been proposed that lower doses of LPS are mainly associated with activation of the MyD88-independent interferon regulatory factor 3-TRIF pathway of TLR4 signaling, leading to induction of anti-inflammatory molecules [134]. Also, in the neonatal rat brain, low-dose of LPS (0.05 mg/kg), which elicits very little microglia and macrophage activation and TNFα production, gives tolerance against hypoxic-ischemic injury, while a higher dose of LPS (0.3 mg/kg) induces increased microglial and macrophage activation and enhanced TNFα expression, resulting in high mortality during hypoxic-ischemia [135]. Thus, acute LPS administration in the neonatal brain induces astrocyte activation and proinflammatory microglial activation that seem to result in the death of oligodendrocyte precursors and dividing neurons, although low doses of LPS may induce anti-inflammatory effects. 

Our previous studies [19] have shown that in contrast to ethanol injection, an intracerebral injection of LPS (1 mg/kg) into P7 mice induces neither significant neurodegeneration nor significant formation of phagocytic microglia 24 h after the injection. However, the number of GFAP^+^ astrocytes increases, as observed in the ethanol-treated brain, although LPS-induced GFAP^+^ astrocytes are more evenly distributed in the cortex compared to ethanol-induced GFAP^+^ astrocytes, which mainly localize in the cortex near degenerating neurons and in the deep cortical layer/white matter region [19]. Thus, neurodegeneration and phagocytic microglial formation are not apparent in LPS-treated samples. In agreement with this, it has been reported that systemic LPS increases IL-1β in Iba1 (a marker for microglia and macrophage)-positive cells, but does not increase CD68 (a marker of phagocytes) in Iba1^+^ microglia [57]. LPS may induce proinflammatory microglia without having morphology of phagocytes. 

In the present study, we systemically administered LPS, ethanol, or both LPS and ethanol into P7 C57BL/6By mice and examined the effects of those compounds on neurodegeneration and glial activation. LPS (0.5 mg/kg, i.p.) or saline was injected 2 h before ethanol/saline treatment (2.5 g/kg, 2 h apart, twice, as described in [11,117]), and 8 h and 24 h after the first ethanol injection, mice were perfusion-fixed and processed for immunohistochemistry as described [117], or the forebrains were processed for Western blot analyses using the Odyssey infrared imaging system as described [136]. We chose two time points, 8 h and 24 h, because it has been shown that P7 ethanol-induced caspase-3 activation peaks around 8 h [11] and morphological activation of microglia peaks around 24 h [19] under the same ethanol treatment conditions. Figure 1A shows the relative amounts of cleaved caspase-3 (CC3) measured by Western blots using anti-CC3 antibody (Cell Signaling) and anti-β-actin antibody (Abcam). The content of CC3 was normalized by actin and expressed as the ratio to the control. The data were obtained using four or five mice (male and female combined) from three different litters per group. The Bonferroni post hoc tests after one-way ANOVA for 8-h samples show that there is a significant (*p* < 0.0001) difference between ethanol and LPS + ethanol groups, indicating that LPS attenuates ethanol-induced caspase-3 activation, although the difference is not significant for 24-h samples. In Figure 1B, P7 mice were treated with LPS and ethanol as described above for Western blot experiments, and 50 µm vibratome sections of the brains from mice perfused 8 h after saline/ethanol treatment were stained using anti-CC3 antibody and Vectastain ABC Elite kit/DAB substrate kit for peroxidase (Vector). The representative images showing the cingulate cortex region indicate reduction in the number of CC3^+^ cells by pre-incubation with LPS. In Figure 1C, brain sections were dual-fluorescence-labeled using anti-Iba1 (Wako) (red) and anti-cleaved tau (tau cleaved by caspase-3, Millipore) (green) antibodies. The representative images show the layers IV/V of the sensory cortex region. Microglia located near cleaved tau^+^ (apoptotic) neurons were morphologically activated, while LPS pretreatment inhibited cleaved tau formation as well as morphological activation of microglia. Thus, previous literature and the present study suggest that while acute LPS treatment in the neonatal rodents causes no or very limited caspase-3 activation or neurodegeneration [19,57,121], it induces proinflammatory reactions [122,123,124]. In contrast to such LPS action, ethanol triggers robust neuroapoptosis in P7 rodents [10,11,12,117] and appears to induce M2-type phagocytic microglia [13,17,19]. Although P7 ethanol may also induce M1-type microglia because transient elevation of mRNA expression of IL-1β and TNFα by P7 ethanol has been reported [13], this proinflammatory reaction seems much weaker compared to that of LPS. However, astroglial activation detected by increased GFAP^+^ cells is found in both LPS and the ethanol-treated neonatal brain [19], and functions of these astrocytes, whether they are neurotoxic or neuroprotective, remain to be elucidated. Also, while LPS alone does not induce significant caspase-3 activation in our experiments, previous studies have shown that LPS induces cell death of oligodendrocytes/oligodendrocyte precursors or inhibits oligodendrocyte differentiation or myelination in the neonatal brain [121,123,124]. Our present study indicates that, similar to the effects of LPS, myelination was affected by P7 ethanol treatment (Figure 2). The expression of myelin basic protein (MBP) analyzed by Western blots as described [136] using anti-MBP antibody (Santa Cruz) decreased 16 h and 24 h after P7 ethanol injection (Figure 2A), and the reduction in MBP expression near the corpus callosum/cingulum area was also observed 48 h after ethanol exposure (Figure 2B). The deficit in MBP expression may be caused by ethanol-induced apoptosis of oligodendrocytes observed in the third-trimester primate brain [137]. Thus, although P7 ethanol-treated mice show apparent quick resolution of microglial activation/neuroinflammation [13,19], there are lingering abnormalities in myelin formation and the number of GFAP^+^ astrocytes [19] that are similar to those induced by LPS treatment. Previously, we and others have reported a reduction in GABAergic neurons in many brain regions in adult mice exposed to ethanol at P7 [25,27,29]. It has been reported that prenatal or neonatal LPS exposure in rodents also induces deficits in GABAergic, especially parvalbumin immunoreactive neurons [138,139,140]. It may be important to examine whether glial activation, even if it is transient, affects long-lasting deficits in cytoarchitecture found in the adult brain. Repeated ethanol administration into the neonatal brain especially appears to induce chronic inflammation [22] and glial cell death [16] as well as long-lasting reduction in several types of neurons [7,24,141,142]. It has been proposed that glial activation observed in acute or repeated ethanol administration in the developing brain may induce the life-long effects because glial cells have important functions during brain development [16]. 

## 6. Conclusions

In summary, activated microglia induced in neonatal mice exposed to ethanol are likely to be neuroprotective as they phagocytose apoptotic neurons. However, the activated microglia can also produce proinflammatory mediators, and prolonged astrocyte activation is observed. Whether glial activation shown in the developing brain exposed to ethanol contributes to cytoarchitectural deficits (such as the reduction in GABAergic neurons) observed in the adult brain remains to be elucidated.
